# Obstetrics and Diseases of Women and Children

**Published:** 1861-07

**Authors:** Wm. B. Atkinson

**Affiliations:** Lecturer on Obstetrics, and Diseases of Women and Children


### OBSTETRICS, AND DISEASES OF WOMEN AND CHILDREN.

BY WM. B. ATKINSON, M.D.,

LECTURER ON OBSTETRICS, AND DISEASES OF WOMEN AND CHILDREN.

### I.—1. *Placenta Prævia.* By D. LLOYD ROBERTS, M.D., Manchester.

Dr. Roberts gives the details of three cases of placenta prævia, which he considers as demonstrative of the fact that turning may be resorted to with safety to the mother, when the os uteri is dilated only to the size of a shilling, provided rigidity be absent; and that waiting for further dilatation, as recommended, may so exhaust the powers of the patient as to render recovery impossible; that the administration of full and decided doses of tincture of opium with Indian hemp, say 90 to 120 drops of the former to 20 of the latter, is beneficial in preventing the syncope which often occurs after severe hemorrhage; that where the child is dead, and version has been performed, and in cases where the os is not sufficiently dilated to permit the easy passage of the head, it is preferable to keep up gentle traction, and trust to the natural powers, rather than to make forcible attempts at extraction and risk a laceration of the os; by the former method the head is brought to press more continuously upon the vessels, thus preventing further hemorrhage.—(*Edinburgh Med. Jour.*, Feb., 1861.)

### 2. *Ibid.* By H. A. MARTIN, M.D., Roxbury, Mass.

Mrs. S., aged fifty-seven, had hemorrhage at the sixth month, which was controlled by the usual means, until the full term had expired, when a pro-

---

fuse discharge suddenly occurred. An examination revealed a firm coagulum blocking up the upper portion of the vagina. As the pains were feeble, and this *tampon* seemed to control the hemorrhage, it was suffered to remain. In about four hours this was expelled, the os was found to be high up, dilated to the size of a half dollar, with two-fifths of the opening occupied anteriorly by the edge of the placenta, behind which the membranes protruded at every contraction. As the hemorrhage was very profuse, and the os unyielding, version was out of the question, therefore the membranes were ruptured, and the vagina carefully plugged with linen; at the same time an infusion of one drachm of the whole grains of ergot in a teacupful of boiling water was given. The pains improved, and in an hour the tampon was expelled; and the os being well dilated, and the child's head well advanced, the forceps were applied, and the delivery at once accomplished; the uterus firmly contracted the moment the placenta was removed. The patient rapidly recovered. The child inspired but once, and no efforts could cause a continuation. Its body presented the appearance of the most complete anæmia, which was evidently the cause of death.—(*Boston Med. and Surg. Jour.*, April 18, 1861.)

3. *Ibid.* By Dr. MARTYN, London.

Mrs. D., at full time, had a very profuse discharge, with very feeble pains, the os dilatable and high up. As she was much exhausted, the hand was used as a tampon, while support and ergot were given. The placenta was then separated from around the os, but fresh hemorrhage coming on, with slight pains, the membranes were ruptured, and the delivery accomplished by version. The child did not respire, and the mother recovered slowly.

Mrs. ——, at the eighth month, hemorrhage profuse. The pains, after an opiate, became stronger, and after a labor of twelve hours, a sharp pain expelled a dead child and the placenta. Recovery slow.

In a third case, near the close of pregnancy, though the hemorrhage was but slight, it was deemed advisable to bring about delivery. The membranes were ruptured, and the placenta detached from the os, with very little loss of blood. But the funis coming down, version was performed, and a dead child extracted. Recovery natural.

Dr. Martyn had met with but nine cases of unavoidable hemorrhage in 1004 labors; one was fatal to the mother and seven to the child.—(*Lancet*, April 27, 1861.)

II.—*The Induction of Premature Labor.* By HENRY JAMES, F.R.C.S., London.

The importance of premature labor, as a means of saving the life of the child as well as of alleviating the sufferings of the mother, induced Dr. James to communicate the method which, for many years, he has followed with almost uniform success. He has since his first operation, in 1848, learned that Dr. Stultz, in 1834, adopted a similar plan.

The patient is placed in the usual obstetric position, and the fore finger of the left hand is passed up to the os uteri, and, as soon as possible, between its lips into the organ; the neck is now to be slightly pulled down, and the finger still within is to be passed round it as far as possible. The passage of the finger is seldom accomplished upon the first attempt; upon the second trial, however, the vagina will generally be found to be more lubricated, and the os lower down; frequently it will be necessary to repeat the manipulation on successive days, a third or fourth time. Great assistance may be rendered by the female bearing down, or by pressure being made with the right hand of the operator upon the fundus uteri. On each occasion some pain will ensue, and ultimately the sensations of labor will come on, when an elastic tube, or a male catheter, attached to an elastic bottle, is to be passed through the os and between the walls of the uterus and the membranes to the extent of from four to six inches. Care is required to avoid rupture of the membranes, and if any resistance is felt, the tube should be withdrawn and passed in another direction. This is done, lest the placenta should be encountered, and thus hemorrhage be produced. The catheter being thus inserted, the elastic bottle is filled with cold water, which by pressure is forced up. Pains follow, and the water is generally returned in gushes. When the os dilates and the membranes protrude, they should be ruptured, and the birth will speedily take place.

Dr. James reports several cases; one woman being delivered four times, three living children; a second, twice, one child living, the mother dying on the second occasion; a third, three times, two living children; the fourth and fifth, twice, one living child each.—(*Lancet*, May, 1861.)

III.—*Thirty-two Years Utero-Gestation.* By J. H. WHITE, M.D., Delaware.

Mrs. R., aged sixty-two, tall, and much emaciated, came under the charge of Dr. White, May, 1860. Had one child by her first husband. At thirty, she fell and struck the lower part of her abdomen transversely on a stick of hewn timber. Severe inflammation ensued, a physician was called, and she was pronounced enceinte. Illness continued, and at the supposed full term, she had severe labor-pains, but without effect. Lactation was shortly established. Falling into other hands, she was treated for dropsy. At this time there was a constant and copious discharge from the vagina of an offensive fluid resembling beef brine. After two years, her health improved, the secretive functions of the uterus were established, and continued regularly till the age of forty-eight. After this she enjoyed comfortable health till sixty, when she commenced to suffer greatly with constant nausea, vomiting, wakefulness, and a desire to urinate, with a bearing down like labor. She passed, during some twenty months of this suffering, small bones and spicula, evidently fœtal. After her death, which occurred in September, an examination revealed in the womb the bones of a fœtus. There were extensive adhesions of the uterus to the lower intestines, pelvis, and bladder, and free communication between the latter and the womb, the mouth of which was closed.—(*Ohio Med. and Surg. Jour.*, March, 1861.)

IV.—*The Etiology of Vomiting in Pregnancy.* By W. M. TURNER, M.D., Petersburg, Va.

Dr. Turner, rejecting all other theories upon the subject, contends that this disorder is a natural affection. The absence of it is due to smallness of the fœtus, to excessive capacity as regards the abdominal, uterine, or pelvic region, or to non-susceptibility. Either of the first two causes would preclude pressure upon the blood-vessels. In the last instance there exists a sluggish state of the brain, etc.; hence there is no general sympathy. This amounts to an inactivity—an unhealthy condition.

Dr. Turner considers the correct cause of morning sickness to be the *pressure exercised on the blood-vessels by the gravid uterus.* Œdema of the lower extremities proves this. Why should not the brain suffer by this irregular circulation? The womb by this pressure we know creates œdema, neuralgia; and it is natural that the sickness should be decided in the morning, after the recumbent posture during the night. The nausea disappears as the patient stirs about, and the circulation becomes improved. In cases where great narrowness exists, the vomiting is more persistent; and, on the contrary, where the fœtus is small, or the pelvis large, there is no vomiting.

The nausea present in ulcerated conditions of the cervix, at menstrual periods, and in malpositions generally, he regards as easily explained by this hypothesis; for generally we find the womb incredibly engorged and enlarged. A woman in La Charité Hospital suffered much with pain in the lumbar regions, and excessive vomitings. By causing her to lie on the abdomen, with the pelvis raised above the level of the head, both affections were relieved as by magic.—(*Amer. Med. Times,* March 16, 1861.)

V.—*Puerperal Fever complicated with Diphtheria, in which Life was saved by the Tincture of Chloride of Iron.* By ROBERT DRUITT, M.D., London.

A healthy woman, aged forty-two, was attacked after labor with shivering, and violent pain in the left leg. This pain soon changed to the left side, below the mamma. There were extreme restlessness, heat of skin, and prostration; and on the third day, violent fetid diarrhœa set in. On the ninth, the motions, which had continued incessantly, were thin, reddish, and passed involuntarily. The patient was drowsy and wandering, had great difficulty in swallowing, and copious salivation, together with a diphtheritic exudation at the back of the fauces. The free use of wine, brandy, beef-tea, opiates, and quinia failed to stay the progress of the malady, and the patient was sinking, when the tincture of the chloride of iron was given, and with decided and unmistakable effects. The evacuations were deodorized, the diarrhœa ceased in twenty-four hours, and, spite of the throat complication, she rapidly became convalescent. The diphtheritic exudation extended over the posterior half of the soft palate. The upper lobe of the left lung was left consolidated, but has since entirely recovered its normal condition. The quantity in which the remedy was given deserves notice; three fluid ounces were swallowed in five days, besides an ounce wasted in lavements. During the first day that it was given, the patient took two fluid drachms every two hours. It is evident that these doses did good, and probably smaller ones might have failed.—(*Obstetrical Society; Med. Times and Gaz.,* Feb. 16, 1861.)

VI.—*Ovarian Disease combined with Pregnancy.* By JOHN HUNTER, M.A., etc., Manchester.

Mrs. W. had been married three years without being pregnant. She suffered with leucorrhœa and pain in the back, which were soon relieved, and menstruation not again occurring, pregnancy was suspected. In about six months she was attacked with severe diarrhœa; which being cured, the abdomen presented the form and size of about seven months' pregnancy; but, except the absence of menstruation, no other symptoms were present. On consultation, the case was diagnosed as one of ovarian disease, and the iodine treatment employed. The patient took five drops of the tincture three times a day, and the abdomen was painted with the remedy and supported by a well-fitting belt. She had scarcely been well under the influence of the iodine, when she passed from the bowels a small cupful of jelly-like matter, which had all the characteristics of the albuminous substance of an ovarian cyst. The treatment was continued, the belt being daily tightened. The evacuations continued, and the tumor grew smaller, when suddenly she was delivered of a dead fœtus of four months. The discharge ceased, she recovered well, and as a slight tumor remained, the iodine was continued. She is now in comparatively good health, and menstruation is regular.—(*Lancet*, Jan. 26, 1861.)

VII.—*Treatment of Galactorrhœa by the Uterine Douche.* By DR. ABEGG, Dantzig.

The sympathy between the womb and mammæ is well known, as also the influence of suckling on the contraction of the uterus. It is on this idea that Scanzoni has founded a new method of producing premature labor by means of an excitement of the breasts. From these facts, M. Abegg has employed the uterine douche in cases of persistent galactorrhœa, and with the greatest success.

He reports two cases, where, from the death of the child in one, and the insufficient development of the breasts in the other, suppression of the milk became necessary. The usual remedies failed, though most thoroughly employed, and he then had recourse to the douche, at the temperature of about 90° F., prolonged for fifteen minutes. This was continued daily for ten days; the secretion was arrested, and the breasts resumed their natural size.—(*Monatschrift für Geburtskunde*, Dec. 1860; *Gaz. Heb.*, Feb. 15, 1861.)

VIII.—1. *Occlusion of the Uterus.* By DR. M. M. PALLEN, St. Louis.

The patient had suffered for two months intolerable anguish, without menstruation, though with no other signs of pregnancy. By the finger in the rectum, a tumor could be felt; a vaginal examination revealed a shortened vagina; three-fourths of an inch above the external orifice there was an opening but no neck of the womb. The uterine sound could not be passed to any depth. Next day, detecting fluctuation in the tumor, a curved trocar was plunged through this supposed os uteri, and a quart or more of menstrual fluid gushed out, relieving the patient.—(*St. Louis Med. and Surg. Journal*, January, 1861.)

2. *Occlusion of the Vagina.* By A. P. HAYNE, M.D., San Francisco.

E. B., aged twenty, was delivered of her first child about one year ago. All went apparently well, though the catamenia never reappeared. Her abdomen was now gradually enlarging, with a sense of fullness and more or less constitutional disturbance. Examination revealed a firm, resisting membranous adhesion, completely closing the vagina. This evidently extended throughout the length of the canal. Dilatation by means of compressed sponge was employed. Strips of sponge, of the width of two fingers, were folded together, placed in the vagina, and retained by a compress and bandage. After twelve hours it was removed, and a fresh strip introduced. By the absorption and dilatation, at the end of six weeks the occlusion yielded, and a sudden gush of the accumulated effusions took place. By a continuance of the method, the vagina was completely restored to its normal size.—(*Pacific Med. and Surg. Journal*, January, 1861.)

3. *Ibid.* By S. L. Abbot, M.D., Boston.

Mrs. ——, aged twenty-five, was found, on examination, to have a transverse diaphragm, obstructing the vagina toward the upper part. It had a circular opening in the centre, but without any characters indicative of an adhesion or contraction of the opposite walls. Pressure upon this caused considerable pain. An operation was advised, but not performed.

Mrs. ——, in labor with her first child, was found to present a complete occlusion of the vagina by an imperforate hymen. The labor was allowed to proceed, and as the head came down the membrane gave way before it, and the delivery was accomplished.—(*Boston Med. and Surg. Journal*, May, 1861.)

IX.—*The Frequency of Conceptions in Anæmia, and some other Constitutional Diseases of Women.* By Dr. E. A. Meissner, Leipsic.

In women who suffer from general disturbance of nutrition, chlorosis, leucæmia, general anæmia, gastric catarrh, etc., conception is disproportionately frequent. Often after an abortion, metrorrhagia, long suppurating mammary abscess, or galactorrhœa, the physicians and relatives of the patient are disagreeably affected by the signs of a new pregnancy. It is also well known how numerous are the offspring among the poor, in the unhealthy, over-populated manufacturing districts, whose inhabitants are generally ill fed. On the contrary, women living under the most favorable external circumstances, free from care, grief, or want, of strong constitution, in full health, are far behind the former in fecundity, conceiving with difficulty and often remaining sterile.

These observations being contradictory to rational supposition, require physiological explanation. General plethora, a plentiful chylopoesis, with a strong nutrition and fat formation produce an abnormal increased pressure of the blood-vessels, and predispose to the phlegmatic temperament and diminished excitability. A more or less indisposition to coitus combined with it may, under certain circumstances, at a high degree of corporeal heaviness, be increased to an almost total antipathy to sexual intercourse. It is a well-known fact that a more lively imagination, increased sexual desire, and heightened voluptuousness are found in nervous, pale, hysteric, and even tuberculous individuals. The irritation of the medulla oblongata and spinalis, based upon the anæmia, keeps up almost constant reflex action, a more excited life in the sexual apparatus, and a disproportionate ovulation, which is often indicated by the too frequent or too strong menstruation not in correspondence with the nutritive condition of the body.

Besides the preponderance of the sexual over the purely vegetative life, the frequency of conceptions in these subjects depends principally upon the more easy and energetic reaction of the same after external and internal stimuli, in conformity with the greater activity, erethismus of their nervous apparatus. Such a high-grade reaction as is not only generally induced by coitus, but also physiologically aimed at as a stimulus of the highest force, is absolutely necessary to favor all the processes of conception. As to the *local* causes, that is, those originating in the condition of the uterus itself, we find this morbid excitement and increase of sexual desire based upon the peculiarity of the sexual life in women, a kind of hunger of the uterus, and which is greater and more lively in *perfect emptiness* of this organ; hence, a deficient nutrition of its muscular structure, small quantity of blood and consequent diminished pressure of the vessels upon its texture.

In direct opposition to this do we find, very frequently, a weak, even abnormally diminished sexual desire in continued or repeated strong determination of blood to the uterus, in consequence of which the volume of this organ is increased.

Again, the form of the womb is essentially and durably modified in habitual congestion, etc., changing from a flat to a rounded form, the external os uteri and canal of the neck becoming very much narrowed. This acts as a considerable obstacle to conception, as proven by sterility being cured by dilating the narrow orifice.

Analogous with these congestions, is the condition of the uterus before and during menstruation, when the sexual desire and the power of conception are much lessened, while immediately after, the faculty of conception is greatly heightened.

In the plethoric there exists a diminished attraction of the sperm in the generative organs; in fact, plethora is an essential obstacle. There is a tendency to exudation, and thus any sperm present would be washed away. Organs empty of blood attract endosmotically the various substances, as serous and albuminous fluids. It cannot therefore appear strange that the anæmic uterus shows a strong disposition to the attraction of the sperm, and hence to conception, particularly after severe hemorrhages, abortions, etc.—(*Cincinnati Lancet and Observer*, March, 1861.)

X.—*Simulated Hip-joint Disease successfully treated by Applications to the Os Uteri.* By DR. STEPHEN SMITH, New York.

Rose, aged twenty-one, was admitted to Bellevue Hospital, suffering for eight years, and treated for hip-joint disease by means of blisters, etc. On careful examination, doubts as to the existence of the disease were entertained; a speculum examination was made, and excoriations were found around the os and cervix uteri. There was some retroflexion and a clear viscid discharge from the os. Applications were made of two drops of chromic acid, once every eight days, to the os uteri, for four weeks. After the second application, she left her bed and walked about, and as the os and cervix were healed, the *hip-disease* diminished. At present the discharge has ceased for three weeks, the excoriations have healed, and nothing but a little soreness in the inguinal region remains.—(*Am. Med. Times*, February 23, 1861.)

XI.—*Vegetations on the Genitals during Pregnancy.* By Dr. Thibierge, Paris.

In 1856, Dr. Thibierge presented a paper on this subject, showing these growths to be solely an accident of gestation, and caused only by it. Many new facts have served to confirm this opinion. It is of great importance to prove that these vulvo-vaginal vegetations are not a syphilitic manifestation, as the majority of physicians believe and the books generally teach. He refers to one case reported by Dr. Zerbe, one by M. Chassaignac, and four by Dr. Ancelet, in all of which there was every reason to believe the truth of the statement, that no syphilis had ever existed; in each the growths resisted all treatment, yet disappeared rapidly and spontaneously with the cessation of the lochia.

Madame X., aged twenty-two, without any syphilitic trace, a short time after the commencement of pregnancy, suffered by a growth of tumors, commencing near the anus, extending above, and rapidly covering the whole perineum. By the third month ulceration occurred to such an extent that the least movement caused extreme pain. The physician, considering them as syphilitic, administered the usual remedies, and cauterized them without success. By the fifth month she consulted Dr. Ancelet, when he found surrounding the anus two very large tumors, extending also upon the vulva, much ulcerated, and discharging freely a bloody, fetid fluid. The pain was very great. He painted the parts thrice daily with tincture of iodine, isolated their surfaces with charpie, and applied powder of peroxide of iron, alum, and savine. This cicatrized the ulcerations, permitted the patient to get about again, but the vegetations persisted. At the eighth month she was delivered of a dead child, with an enormous cephalæmatome. A month after the delivery the vegetations had spontaneously disappeared.

Another case, very similar in its history, obstinately resisted all caustics, and left the hospital, without the slightest improvement, at her second month of pregnancy.

After giving the details of a number of similar cases, he relates one where syphilis was present, and the vegetations continued, though by mercury, etc. the syphilitic symptoms were soon dissipated. Here, as is occasionally seen, they persisted even after the delivery, requiring only a local treatment for their cure.

In the great majority of cases, he prefers the application of the acid nitrate of mercury, which causes their disappearance in about four days. Although this treatment is generally safe, yet it should be known that occasionally it gives rise to accidents. In one case it produced mercurial stomatitis.

This subject has proved of much importance, since it may become necessary to decide in a medico-legal view as to whether these vegetations are the result of syphilis or merely of pregnancy. Dr. Louis Penard, in a recent publication, gives an opinion in such a case. A young girl, pregnant six months, has a muco-purulent discharge, and at the orifice and in the cavity of the vagina vegetations of various sizes. His opinion was that nothing existed to cause the belief in a syphilitic origin, as she had none of the symptoms of syphilis, no affection of the skin, no ulcerations; in short, the tumors are due solely to the fact of her being pregnant, and the leucorrhœa has a similar origin in conjunction with the peculiar constitution of the patient.

In conclusion, Dr. Thibierge recommends having recourse to surgical treatment only when the vegetations are very large and persistent.—(*Gaz. Hebdom.*, February 8 and 15, 1861.)

### XII.—*Puerperal Vaginitis.* By M. BEAU, Paris.

M. Beau mentions a form of vaginitis with a discharge strongly resembling that of gonorrhœa, and which is the result of pregnancy. It is of common occurrence, and obstinately resists treatment. Nitrate of silver, in solution, applied freely, is the best application.—(*Gaz. des Hôpitaux*, No. 147, 1860.)

### XIII.—*Uterine Phlebitis and Puerperal Inflammations.* By Dr. SEBASTIEN.

Dr. Sebastien, having been very successful in a large number of obstetric cases, both in private and hospital practice, attributes his success to the employment of

℞.—Potas. nitrat. p. j;

Tinc. arnica, p. iv;

Syr. acac. p. l;

Aquæ, p. ccc.—M.

Dose, a tablespoonful every hour.—(*Gaz. des Hôpitaux.*)

### XIV.—*Prolapsus of the Posterior Wall of the Vagina; Operation successful.* By DR. T. G. RICHARDSON, New Orleans.

This case, a negro woman, aged fifty, was admitted to the Infirmary with a supposed prolapsus uteri, of many years standing, the result of frequent child bearing. A soft, reddish tumor was visible between the labia, and upon separating the parts, it was found to be composed of the mucous membrane of the posterior wall of the vagina. The womb was also depressed, but not to such a degree as to constitute prolapsus, as the displacement was seemingly the result of the relaxed condition of the vaginal wall. The patient's health being good, after the usual preparation, Dr. Richardson operated by removing a large V-shaped flap of mucous membrane from the prolapsed wall, and united the edges by means of silver sutures. She was kept on her back, the bladder regularly emptied by a catheter, and the bowels confined by the use of opium. The sutures were removed by the eighth day, and union was found to be complete. Twelve months after the operation, the patient remained perfectly well. —(*Report of Stone's Infirmary, New Orleans.*)

### XV.—*Will a Child born after the Mother has had Small-pox, and contracted after she has conceived, be liable to contract the Disease?* By ARCHIBALD HALL, M.D., Montreal.

This paper is intended as a partial reply to a similar question in another journal.

Dr. Hall was called to a patient, aged eighteen, pregnant six months with her first child, laboring under varioloid. Symptoms of approaching labor were relieved by morphia, and she passed favorably through the attack. At full time, she was delivered of a healthy boy, who exhibited no signs of having suffered by its mother's sickness. When a month old, vaccination was twice attempted, but failed. Dr. Hall attributes the failure "to the protective influence afforded by the mother's blood, when circulating through the infant's system during its intra-uterine existence, and while the mother was suffering under the disease, operating upon the constitution of the child, and producing its effects precisely as it is doing on the constitution of the mother. We cannot, of course, explain how this protective agency is exerted, although we can appreciate the positive existence of such a preventative or protective influence in its effects, and I feel bound to consider in this instance the protective influence of the attack of variolous disease on the mother prevented the impregnation of the infant's system, exactly as it would have done in the mother herself."

The child did not exhibit any cicatrixes on any part of its body, and Dr. Hall asks, "Could it have had the disease in utero, and the formation of the ordinary pock-marks have been prevented by the continual application to its surface of the liquor amnii? My own opinion is, that it had not the disease, or I should in all probability have had a case of premature labor to manage, as the consequence of its death. But if it had had it, it would have been a convincing proof of the truth of the theory, that to prevent pitting in small-pox, we should exclude the pustules from all contact with the air."

A precisely similar case was related to Dr. Hall by Dr. Stranaghan. Dr. Hall seems too much inclined to reason on the *post hoc, propter hoc* method, for though his theory has a probability in its favor, yet as it has fallen to the lot of the writer to see many instances where, under highly favorable circumstances, vaccinea has failed to be developed, even though the parent has not previously suffered with any disease, he cannot see the force of the argument as applied in these two cases.

Again, when alluding to the probability that the cicatrixes were prevented by the exclusion from the air, he would seem to forget the fact that many instances are on record of infants born with the whole body disfigured by the pock-marks; yet these children were not less excluded from contact with the atmosphere. We should be strongly inclined to regard both cases as mere coincidences, and that the children belonged to that small class of persons who seem to be proof against all kinds of poisons.—(*Brit. Am. Jour.*, February, 1861.)

2. *Ibid.* By A. H. David, M.D., Montreal.

Dr. David relates a third case, eight months pregnant, so severely attacked as to be delirious and very ill. The twelfth day of the eruption, labor came on, and she was delivered of a fine healthy girl, without any spots or marks of any kind. At the age of three months vaccination was twice attempted and failed, and having gone to England, it has failed there also.

During a large midwifery practice, Dr. David has hitherto invariably found small-pox to produce miscarriage in pregnant females, and the children were still-born, covered with the eruptions. "These cases may become of importance, on the question of vaccination and revaccination, of which we still have a great deal to learn."—(*Ibid.*, March, 1861.)

XVI.—*Treatment of Prolapsus Ani of Children by Subcutaneous Injection of Sulphate of Strychnia.* By M. FOUCHER, Paris.

In the case related, the child, aged four years, had suffered for several months; when reduction was not immediately accomplished, the part was closely grasped by the sphincter, producing great pain. M. Foucher, with one of Pravaz's syringes, injected, externally to the anus, but in the direction of the fibres of the sphincter, ten drops of a solution, in the strength of one part by weight of strychnia to one hundred parts of water. Twenty-four hours after, fourteen drops were injected. The cure was then complete and permanent.—(*Gaz. des Hôpitaux*, No. 83.)

